# Adolescent mental health in rural settings: the role of artificial intelligence and community engagement

**DOI:** 10.3389/fpubh.2025.1643466

**Published:** 2025-09-04

**Authors:** Sarah Grenon, Khanh N. Hoang, Si Luo, Amie Koch

**Affiliations:** School of Nursing, Duke University, Durham, NC, United States

**Keywords:** rural health, adolescents, mental health, artificial intelligence, community engagement

## Abstract

Adolescents in rural communities face persistent mental health disparities due to provider shortages, social isolation, and stigma. Traditional care models remain insufficient. This perspective explores how artificial intelligence (AI) tools combined with community engagement strategies can enhance adolescent mental health support in underserved settings. A targeted literature review identified best practices and innovative models that integrate AI in culturally relevant ways. Findings highlight the importance of trusted community partnerships and digital literacy, particularly prompt literacy, in ensuring safe and effective AI use. Nurses are uniquely positioned to lead these efforts, promoting health equity and digital inclusion through community-based, AI-enabled interventions.

## Introduction

Adolescent mental health challenges represent an urgent public health concern, particularly in rural communities where persistent disparities are fueled by provider shortages, geographic isolation, and stigma. Nearly 30% of rural high school students report feeling persistently sad or hopeless, with suicide rates among rural teens (16 per 100,000) exceeding urban rates by 70% (1). These statistics highlight the inadequacy of traditional care models and the need for innovative approaches.

**Figure 1:**
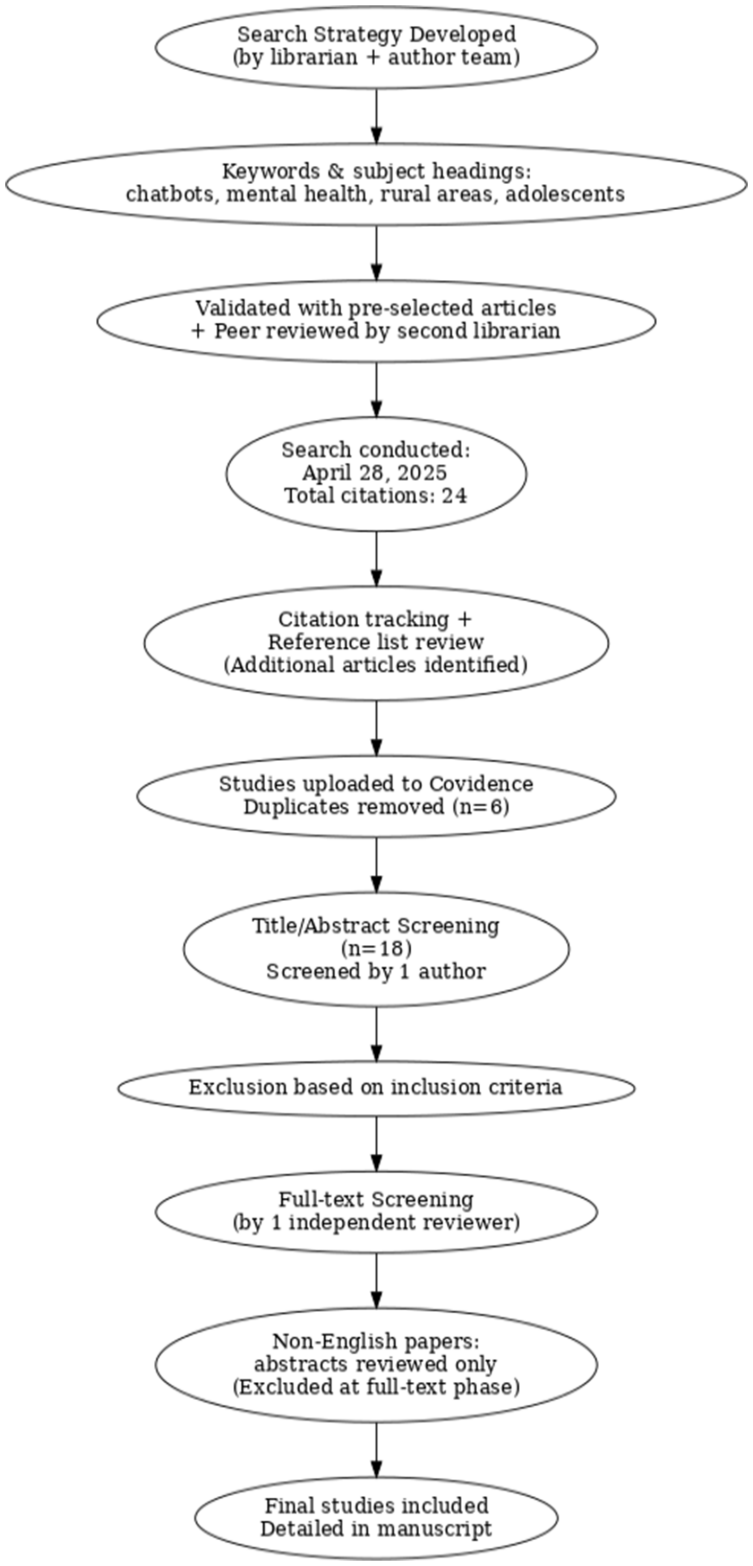
Literature review process.

This manuscript explores how artificial intelligence (AI) technologies can be paired with community engagement strategies to support rural adolescents more effectively. AI models can detect mood changes through writing patterns or wearable device data, enabling timely, personalized responses. A targeted literature review identified best practices for applying AI in developmentally appropriate and culturally responsive ways (Figure 1).

When implemented through trusted community partnerships, AI tools can expand mental health intervention reach and relevance. Nurse-led, AI-enabled, community-based strategies hold particular promise for advancing mental health equity in underserved settings by equipping trusted adults with basic training in mental health support and ethical AI use.

## Background

Youth in rural communities face disproportionately high rates of anxiety, depression, behavioral challenges, and suicide compared to their urban peers. For example, in a study of semi-rural high school students in Georgia, 43% reported symptoms of anxiety and 55% reported symptoms of depression (2). These mental health disparities are exacerbated by a chronic shortage of resources, more than 60% of federally designated Mental Health Professional Shortage Areas (MHPSAs) are located in rural or partially rural regions (3). As a result, rural adolescents often wait weeks or even months for services, if they are available at all (4). The combination of adolescent developmental vulnerabilities and fragmented support systems places youth at increased risk for untreated mental health conditions. Alarmingly, only one in five rural adolescents with a major depressive episode receive treatment, compared to nearly half of their urban counterparts (5), highlighting the urgent need for integrated, community-based care models.

Stigma further deepens these disparities. In close-knit rural communities, concerns about confidentiality and social judgment often deter families from seeking professional mental health support. Misconceptions about mental illness persist, and seeking care may be perceived as a personal failure or weakness. As a result, adolescents frequently turn to informal support networks, such as family members, peers, or faith leaders, who may be trusted but are not trained to manage complex mental health needs (6). This reliance on untrained supports can delay or prevent effective intervention, contributing to prolonged distress or worsening symptoms. Reducing stigma requires culturally sensitive, community-informed strategies that normalize help-seeking, build mental health literacy, and empower trusted local figures with foundational training. Strengthening these efforts is essential for creating accessible, developmentally appropriate, and sustainable pathways to care for rural youth.

Community engagement is a vital part of the solution. Successful models, such as school-based mental health programs, partnerships with congregational nurse initiatives, and youth mental health first aid training in rural churches, demonstrate that culturally relevant, community-rooted approaches can improve access and outcomes. These partnerships promote trust, reduce stigma, and help bridge gaps in service delivery.

To respond to these pressing needs, this manuscript aims to: (1) identify the unique mental health challenges rural adolescents face; (2) examine the role of community engagement in addressing these challenges, highlighting programs that have demonstrated success in rural settings; and (3) propose innovative, evidence-informed strategies to enhance access to care for rural youth through collaborative and scalable interventions.

## Methods: information sources and search strategy

To guide this perspective, a targeted literature review was conducted using a structured and peer-reviewed search strategy focused on the use of AI in rural adolescent mental health. Given the interdisciplinary complexity of this topic, the author team collaborated with a professional medical librarian to ensure the development of a methodologically sound and comprehensive approach. This early collaboration was also intentional, as the authors plan to expand this work into a full systematic review in the future. The librarian’s expertise provided a strong foundation for both the current targeted review and future work.

Three databases were selected based on their relevance to healthcare, nursing, behavioral sciences, and mental health: Ovid MEDLINE for its broad coverage of biomedical and clinical literature, CINAHL Complete (via EBSCOhost) for its emphasis on nursing and allied health fields, and APA PsycINFO (via EBSCOhost) for its comprehensive indexing of psychological and behavioral health research. This combination ensured appropriate breadth and focus, capturing literature at the intersection of adolescent mental health, rural health, and AI-enabled interventions.

The final search strategy, developed and executed by the medical librarian, included both controlled vocabulary (e.g., MeSH in MEDLINE and CINAHL Headings) and relevant free-text keywords. The search was independently peer-reviewed using the PRESS (Peer Review of Electronic Search Strategies) checklist and validated against a pre-identified set of relevant articles to ensure sensitivity and coverage. The search, completed in April 2025, retrieved 24 citations. Records were imported into Covidence, and duplicates (*n* = 6) were removed through both automated and manual review. Complete, reproducible search strategies, including database names, exact search strings, filters, and date ranges, are available in the Supplementary Materials. Reference lists of included articles and citation tracking (via Google Scholar and Scopus) were also used to identify additional studies relevant to this review.

### Inclusion and exclusion criteria

To guide the targeted literature review, we used clear inclusion and exclusion criteria. The inclusion criteria for this review are as follows: primary studies involving adolescents aged 12 to 19 years; studies that examine AI-powered interventions such as chatbots, virtual agents, or machine learning applications; and studies focused on rural, remote, underserved, or resource-limited populations. Eligible studies must address mental health outcomes or crisis intervention and be published between 2019 and 2025 to reflect the rapid development of AI technologies. Pilot studies, feasibility studies, and implementation research are included. These exclusions are listed in the appendix. We also excluded studies that did not present original research, such as commentaries or opinion piece, as well as those that focused only on adults or urban populations. Other excluded articles either did not describe the intervention clearly or were based on general health education chatbots that did not use AI. Studies with inaccessible full texts were also excluded. After removing duplicates, 18 articles remained for title and abstract screening, which was completed by one author. Full-text screening followed, and any studies that did not meet the inclusion criteria were removed at that stage.

## Community engagement

Training the trusted adults in adolescents’ lives, such as teachers, school counselors, coaches, faith leaders, and community nurses, is essential for the safe and effective use of AI in youth mental health support. These individuals are often the first to notice when a young person is struggling, particularly in rural areas where access to mental health services is limited. Providing these stakeholders with foundational training in prompt literacy, digital safety, and mental health awareness equips them to guide adolescents in navigating AI tools in ways that are safe, supportive, and developmentally appropriate. According to the World Health Organization (WHO), community engagement involves cultivating relationships that empower stakeholders to work together toward shared health goals, emphasizing mutual responsibility and collaboration as key drivers of improved health outcomes (7).

By involving community members in the planning, implementation, and evaluation of mental health programs, community engagement ensures that these initiatives are tailored to the specific needs of the community, thereby increasing their effectiveness and sustainability.

The Community as Partner model reinforces this approach by recognizing that health is shaped not only by clinical care, but also by social, environmental, and relational factors. It promotes a participatory framework where stakeholders are actively engaged throughout all stages of a program, from identifying needs to co-developing and implementing solutions. For example, a school counselor familiar with mental health chatbots can support students in crafting thoughtful prompts, knowing when to seek in-person help, and using AI tools to enhance self-reflection and well-being. This shared digital fluency fosters trust and deepens the informal support systems already present in young people’s lives.

### Community as partner model

At the heart of the Community as Partner model is the engagement of community stakeholders who already have strong, trusting relationships with adolescents. Educators, clergy, public health nurses, and youth mentors are often the first adults young people turn to when facing mental health challenges, particularly in rural communities where formal services may be limited or stigmatized. When these trusted figures are equipped with the right tools and training, they are well positioned to support youth in meaningful and culturally relevant ways. By collaborating with individuals, families, local organizations, and key stakeholders, community engagement helps build trust, mobilize resources, and promote sustainable solutions tailored to community needs. This collaborative approach strengthens mental health literacy, reduces stigma, and enhances the cultural relevance of services, ultimately improving their accessibility and acceptability (7, 8).

Community-based workshops or train-the-trainer programs focused on AI literacy, prompting techniques, and youth-centered mental health communication can prepare these adults to confidently integrate AI tools into their everyday interactions with teens. For instance, rural churches might include digital mental health training for youth ministers, while after-school programs could offer AI-guided emotional check-ins led by staff. Providing basic training in digital safety, mental health awareness, and how to ask reflective, supportive questions enables community members to guide adolescents in using AI-powered mental health resources safely and effectively. This approach not only expands access to support but also ensures that interventions are embedded within familiar, trusted community structures, making them more sustainable and responsive to local needs.

Suppose a rural community pilots a program to support adolescent mental health using AI-enabled tools, in partnership with school nurses, youth ministers, and 4-H leaders. Recognizing that many teens in the region were hesitant to speak with clinicians due to stigma, the program leveraged the trust students already had in community adults, particularly those in schools and faith-based settings. Through a series of community-based workshops, these adults received training in prompt literacy (learning how to ask emotionally attuned questions using tools like Wysa and Woebot), ethical AI use (including guidance on privacy, consent, and avoiding misinformation), and basic mental health first response. Youth mentors were also equipped with sample prompts, such as “What are healthy ways others manage social pressure?” to use in conversations with teens. Over 6 months, mentors reported feeling more confident in addressing mental health concerns, while students became more open to using AI tools, describing the experience as less judgmental and more approachable when introduced by someone they trusted. This program shows how integrating digital tools into trusted community relationships can turn familiar support systems into powerful pathways for connecting teens to mental health support.

This approach builds on proven models like the Youth Aware of Mental Health (YAM) program, a school-based initiative designed to help adults lead meaningful conversations with teens about mental health. YAM has been used in both rural and urban settings and has shown real impact. In a large study involving around 11,000 ninth-grade students across ten European countries, students who participated in YAM were more than 50% less likely to act on a suicide plan compared to those who did not take part. By embedding this kind of support into relationships that adolescents already trust, we can help make digital mental health tools more approachable, relevant, and effective. When paired with AI-enabled tools, such as chatbots, mood tracking apps, or wearable sensors, programs like YAM could offer even greater impact by extending support beyond the classroom, offering timely, personalized interventions that reinforce what’s taught in person.

Further illustration of successful community-based mental health intervention include the Live4Life program in Ballarat, Australia, and the school-based mental health clinic in Southside Independent School District (ISD), Texas. Both programs highlight how locally grounded efforts can make a real difference in adolescent mental health, especially in rural communities. In Ballarat, more than 1,200 students took part in Teen Mental Health First Aid, and nearly 300 adults, including school staff and community members, received training in how to support young people. Just months after the program launched, 73% of students said they could recognize when a peer might be struggling, 88% had already reached out to someone for help, and over 80% said mental health education in school is important. These results echo similar outcomes from earlier versions of the program in other regions, where young people reported having more conversations about mental health and adults felt more confident stepping in when needed.

In Southside ISD, a new partnership with Clarity Child Guidance Center brought mental health care directly to students through the district’s first on-site clinic. Even before the clinic’s official opening, it had already served seven students and families. In an area with no local psychiatrists and few mental health resources, the clinic now offers therapy, counseling, and psychiatric care right on campus. With about 6,000 students in the district, this makes support much easier to access, especially for families facing transportation or cost challenges. Services are covered for Medicaid-eligible students, and a sliding fee scale ensures no one is turned away. Together, these programs show how community involvement and thoughtful partnerships can lead to more accessible, meaningful, and lasting mental health support for young people.

## Evidence-based treatment modalities

Anxiety disorders, depressive disorders, and ADHD are among the most common mental health conditions affecting adolescents (9). Recent data indicate that among U.S. individuals aged 3–17 years, 9.4% have been diagnosed with anxiety, 4.4% with depression, and 9.8% with ADHD (10). These conditions frequently co-occur and are characterized by symptoms such as low mood, excessive worry, difficulty with attention, hyperactivity, and impulsivity, which can significantly impair various aspects of life. If left untreated, these conditions can lead to severe psychopathology and health-related issues in adulthood, making their effective treatment a critical public health concern with implications across the lifespan (9).

Fortunately, evidence-based treatments are available. Behavioral therapies, such as parent training, are effective for managing ADHD, while cognitive behavioral therapy has shown efficacy in treating anxiety and depression in youth. Medications, including stimulants and nonstimulants for ADHD and selective serotonin reuptake inhibitors (SSRIs) for anxiety and depression, have also demonstrated therapeutic benefits.

Unfortunately, there is limited accessibility to trained child and adolescent psychiatrists for the prescribing of pharmacotherapy, especially in rural areas. Additionally, some evidence-based pharmacotherapies are controlled substances (e.g., stimulant medications) that require regular in-person visits, which may not be feasible for patients and families.

The implementation of evidence-based mental health treatment for adolescents is increasingly being delivered in accessible, community-based settings, including schools, homes via telemedicine, faith-based organizations, mobile clinics, and peer-led initiatives. These interventions aim to increase accessibility, reduce stigma, and provide early identification and treatment for mental health concerns.

### School-based mental health

School-based mental health programs are effective in reaching adolescents who may not otherwise access mental health services. For example, the Health Resources and Services Administration (HRSA) awarded nearly $25 million to improve and strengthen access to school-based health services in underserved communities (11). These programs provide comprehensive primary health care services, including mental health support, directly within schools, making mental health services accessible for school students. However, this model of relying on HRSA grants is not a sustainable model for continuing care if funding is not secured.

The good news is that there are fiscally stable, sustainable solutions available to support school-based mental health programs, and many of them can effectively integrate AI to enhance care. Medicaid reimbursement, including School-Based Health Services (SBHS) offers ongoing funding for eligible mental health services, which can be supported by AI tools for screening, documentation, and triage. Partnerships with Federally Qualified Health Centers (FQHCs) also provide a reliable funding stream, enabling services to be delivered on-site or through telehealth, with AI assisting in early identification and referral. In addition, state mental health block grants, nonprofit hospital community benefit programs, and blended funding models between education agencies and mental health authorities offer additional opportunities for sustainable investment. Public–private partnerships can further offset costs and expand access to AI-enabled tools such as chatbots and mood tracking apps. Together, these strategies offer promising pathways to create lasting, tech-enabled mental health support for adolescents in schools, especially in underserved areas.

### Telemedicine

Recent studies show that telehealth is an effective way to treat anxiety and depression in adolescents, with outcomes similar to in-person therapy. Remote delivery of cognitive behavioral therapy (CBT), in particular, has been shown to reduce symptoms and improve daily functioning. Telehealth also helps eliminate common barriers, like transportation issues or the stress of attending in-person appointments, making it especially helpful for youth in rural areas (12).

AI can add even more value by helping to personalize and extend the reach of telehealth services. For example, AI tools can monitor mood patterns, guide adolescents through CBT-based exercises, and offer real-time support when a therapist is not immediately available. These tools can help keep adolescents engaged between sessions and alert providers if symptoms worsen.

However, limited internet access and the cost of digital devices continue to be major barriers, especially for vulnerable and socially disadvantaged groups in rural communities. Addressing these gaps will require strong policy support, investment in broadband infrastructure, and a commitment to making digital tools affordable and accessible for all families.

### Behavioral health and primary care

Integrating behavioral health into primary care settings can significantly improve access to mental health services for adolescents. One widely adopted approach is the Screening, Brief Intervention, and Referral to Treatment (SBIRT) strategy, which facilitates early identification and support for substance use and related risks in pediatric care SBIRT helps maintain continuity of care by reducing fragmented services and wait times, making it particularly valuable in rural settings with limited specialty mental health resources. AI can enhance this model by automating electronic screenings (e.g., flagging high-risk responses in real time), providing decision support for brief interventions, and aiding in follow-up monitoring. These AI-enabled systems not only streamline workflows and improve risk detection but also ensure timely follow-up, helping to prevent adolescents from falling through the cracks.

### Faith-based programs

Faith-based counseling weaves spiritual support into mental health care—especially meaningful in rural areas where churches often serve as community hubs. To enhance these efforts, AI tools like mental health chatbots and AI-guided journaling apps can offer teens a private, nonjudgmental space to reflect on emotions and build coping skills. Apps such as Reflectly, Journey, and Daylio use AI to personalize reflections and track mood patterns over time, while others like Rosebud, Clearful, and Juniper provide guided prompts, entry analysis, and AI-generated insights to help users better understand their emotional experiences (13).

A powerful complement to these tools is the Empower trainings from Gateway to Hope University, which prepare lay leaders, including faith leaders, teachers, and volunteers, to recognize signs of emotional distress and respond with compassion using the 4 R’s framework: recognize, relate, respond, and refer (13). By combining AI-supported journaling with in-person support from trained, trusted adults, communities can create a more inclusive and responsive ecosystem for adolescent mental health, particularly for LGBTQ+ youth who may face stigma in some religious settings. This blended approach helps make emotional support both accessible and affirming.

### Community-based programs

AI has the potential to significantly enhance community-based mental health programs like Virginia’s REACH initiative by improving efficiency, personalizing support, and expanding access, particularly in rural areas with limited resources (14). For example, AI-powered triage tools can analyze language patterns from crisis calls or messages to flag high-risk situations in real time, helping teams prioritize and respond more effectively. Predictive analytics can also be used to identify individuals at increased risk of a mental health crisis based on behavioral trends, previous service use, or wearable data, allowing for earlier intervention. AI chatbots, such as Wysa or Woebot, can offer immediate emotional support before a crisis team arrives, helping to stabilize individuals during the critical waiting period. In addition, AI can automate documentation and follow-up tracking, reducing administrative burden and improving continuity of care. Tools that analyze community-level data can help identify crisis hotspots, enabling more strategic deployment of mobile units. AI translation and voice tools also support more inclusive care by improving communication with individuals who speak different languages or have communication barriers. By integrating these technologies, programs like REACH can deliver more timely, personalized, and accessible mental health care where it’s needed most.

### Mobile health clinics

Mobile health clinics play a critical role in delivering on-site mental health assessments and connecting individuals in crisis to ongoing, longitudinal care. These mobile units often offer a public health–oriented approach to mental health intervention, providing services such as cognitive behavioral therapy (CBT), suicide prevention counseling, substance use screening, and case management (15). In rural communities, mobile clinics bring essential care directly to schools, homes, or community centers, reducing both logistical and stigma-related barriers.

AI technologies can further strengthen the reach and efficiency of mobile mental health services. For example, AI-powered tools can assist with triage by analyzing initial intake responses or symptom screeners to prioritize urgency and match patients to appropriate care pathways. Mood tracking apps and chatbot companions can be deployed before and after visits to provide ongoing emotional support, gather symptom data, and alert clinicians to changes in mental health status. In mobile settings, AI-enabled decision support tools can also help clinicians make faster, more informed assessments during short on-site visits. Additionally, AI can be used for predictive analytics, helping programs identify patterns in community mental health needs and deploy mobile clinics more strategically based on geographic or population-level trends. By combining the accessibility of mobile clinics with the personalization and scalability of AI tools, communities can deliver more responsive, data-informed, and continuous mental health support to underserved populations.

### Youth community engagement initiatives

Community-level approaches to supporting youth mental health and resilience rely on cross-sector collaboration among schools, healthcare providers, local organizations, and families to create environments that foster well-being and reduce risk. These initiatives often prioritize youth leadership and active participation in the design, implementation, and evaluation of mental health programs, ensuring that interventions are both relevant and empowering. This type of community engagement is particularly impactful in rural areas, where traditional mental health resources may be limited or difficult to access, and where trust in local relationships plays a vital role in care-seeking behaviors (16).

AI can be a valuable tool in these community-driven efforts by helping to gather real-time insights into youth needs and preferences. For instance, AI-powered surveys or sentiment analysis tools can analyze aggregated, anonymous responses from students or youth groups to detect emerging trends in stress, anxiety, or social dynamics—allowing communities to tailor interventions more effectively. AI can also support youth-led initiatives by offering platforms for digital storytelling, mood tracking, or project design, giving adolescents accessible ways to contribute data and shape programming. Furthermore, AI-based collaboration tools can help coordinate efforts across sectors, streamline communication, and monitor progress on shared goals. When combined with youth engagement and community partnerships, AI offers a promising way to enhance the responsiveness, inclusivity, and impact of local mental health initiatives.

## Ethical considerations and safeguards

When implementing AI-driven mental health tools for rural adolescents, ethical considerations must be central to design and deployment. Data privacy and security are especially critical in close-knit rural communities, where maintaining anonymity can be challenging. Technical safeguards such as end-to-end encryption, local data processing, automatic data deletion, and balanced parental consent protocols are essential. Community-specific practices should also be in place, including clear policies for information sharing, well-defined crisis response protocols that protect privacy, and confidentiality training for local partners. Additionally, AI systems risk perpetuating algorithmic bias if trained on datasets that do not reflect the lived experiences of rural or diverse populations. To promote fairness, it is vital to incorporate rural perspectives into training data, conduct regular bias audits informed by community feedback, and include cultural competency reviews of AI-generated responses. Transparent reporting of system limitations is also necessary. Finally, addressing the digital divide is crucial for equitable access. This includes developing offline-capable tools, offering device lending programs through schools or libraries, ensuring multi-modal access to accommodate varied user needs, and providing affordable or free services for low-income families.

## Discussion

Innovative interventions that leverage AI offer promising avenues for bridging these gaps through personalized support, early detection, and expanded access to resources. AI-powered tools, such as mental health chatbots, predictive analytics, and virtual agents, can complement traditional services by reaching youth in remote areas, facilitating real-time engagement, and supporting overburdened health systems (17, 18). As digital health technologies evolve, the thoughtful integration of AI into adolescent mental health strategies will be increasingly vital to addressing disparities and improving outcomes in marginalized populations. Table 1 emphasizes the opportunities to use innovative ways to integrate AI into adolescent mental health therapy that are unique to this population. These methods complement conventional therapeutic modalities by integrating culturally relevant, experiential, and technology-enhanced strategies. Table 2 summarizes five such approaches that have demonstrated positive outcomes in research and practice, offering promising avenues for youth engagement and retention in mental health services.

**Table 1:** Innovative enhancements to traditional therapy for adolescents.

Intervention type	Description	Cited benefit/reference
Creative arts therapies	Use of music, art, or drama to facilitate emotional expression and healing.	Reduces stress, improves self-awareness (11, 23).
Adventure therapy	Therapeutic outdoor activities that promote growth and social skills.	Improves self-concept and behavior (23).
Hip-Hop therapy	Engages youth through rap, lyric writing, and cultural expression.	Builds rapport and supports trauma recovery (24).
Virtual reality (VR) therapy	Immersive virtual experiences for exposure and emotional regulation.	Reduces stress and anxiety (25).
Gamified therapeutic tools	Interactive games embedded with cognitive-behavioral principles.	Enhances motivation and therapy adherence (26).

**Table 2:** Innovative AI interventions (27–49).

Intervention	Function in adolescent mental health support
Community engagement	Establishes trust and tailors services to local needs
AI Tools (e.g., chatbots and analytics)	Extend access and assist with early detection of distress
Digital safety education	Teaches youth and adults how to use technology safely and effectively
Cultural relevance	Ensures AI interactions and mental health content are acceptable and inclusive
Health equity outcomes	Improves access and quality of care in underserved rural areas

### Prompting literacy

The skill of prompt literacy, which is the ability to craft effective and context-aware questions for AI tools, supports meaningful and safe therapeutic engagement. In this context, prompting serves not only as a technical function but also as a therapeutic gateway (19). The way questions are phrased sets the tone and intention, while the way users respond to AI-generated content influences how relevant, empathetic, and safe the interaction becomes. Just as young people learn to think carefully about their media consumption and the friendships they build, they will also need support in how to engage meaningfully and safely with digital mental health tools. Developing this discernment includes learning to ask reflective and emotionally sensitive questions, steering conversations toward insight, and recognizing when a live mental health professional should be involved. Instead of relying on rigid scripts where a symptom always leads to a diagnosis, prompt literacy encourages a more flexible and thoughtful approach. This method invites users to explore patters, context, and emotional nuance.

For younger adolescents (ages 12–14), prompting strategies should be concrete and scenario-based. For example, a helpful prompt might be, “Can you help me understand why I might feel nervous before a test and what other kids do to feel better?” This avoids overly abstract or diagnostic language like “Analyze my anxiety patterns,” which may be confusing or unproductive for this age group.

For older adolescents (ages 15–19), prompting can encourage more self-reflection and pattern recognition. A suitable prompt might be, “I’ve noticed I feel more anxious on Sunday nights. What might be contributing to this pattern, and what are some evidence-based strategies that might help?”

Sample prompting protocols further illustrate this concept. For stress management, a more productive prompt is, “I’ve been feeling overwhelmed lately. Can you help me explore what might be contributing to this feeling and suggest some coping strategies that have worked for other teens?” rather than the less effective, “Do I have anxiety?” For social difficulties, instead of asking, “Why do not people like me?” a better prompt might be, “I’m having trouble connecting with peers at school. What are some ways other teenagers have successfully built friendships, and what social skills might be helpful to practice?”

### Enhanced telehealth services

Telemedicine has already demonstrated success in improving access to mental health services for rural adolescents, its future will likely include deeper integration. Wearable devices, such as smartwatches, fitness trackers, and biosensors, can detect physiological changes like elevated heart rate or disrupted sleep, providing early warning signs of stress or anxiety. When paired with AI platforms, this data can prompt timely check-ins or connect users to supportive content or human responders. However, this innovation raises serious privacy and data security concerns, especially when working with minors. Policies must address data ownership, parental consent, and safeguards against commercial misuse or algorithmic bias (20).

Next,-generation telehealth platforms may incorporate AI-driven triage tools to prioritize urgent needs and ensure continuity of care through intelligent scheduling and follow-up mechanisms. Personalized dashboards could give providers access to mental health trends based on passively collected data, helping them to deliver care tailored to each adolescent’s unique circumstances. Importantly, AI can support remote screening, progress tracking, and real-time emotional analysis via voice tone, facial expression, and typing patterns that further close the accessibility gap in resource-limited areas (21). As these technologies evolve, the approach to mental health assessment may also shift from rigid, symptom-based models to more adaptive systems that account for context and individual circumstances. This shift aligns more closely with the complexity of adolescent development and mental health trajectories. Probabilistic AI systems can synthesize risk profiles across behavioral patterns, environmental stressors, and peer interaction trends to predict mental health needs before crises emerge (22). While these models may enable earlier and more nuanced interventions, they demand careful governance to avoid bias or over-pathologizing typical adolescent experiences.

## Combining traditional therapy with AI-powered tools

AI-powered tools such as chatbots, virtual mental health therapists, and predictive analytics tools are becoming increasingly sophisticated and contextually responsive. Platforms like Woebot, Wysa, and Replica have already shown promise in reducing symptoms of depression and anxiety among adolescents, particularly when stigma or provider shortages create barriers to care (21). Recent evaluations of digital mental health tools like *Woebot* and *Wysa* show how these platforms can support adolescents, particularly in settings with limited access to traditional therapy. In a 2023 randomized controlled trial, *Woebot for Adolescents (W-GenZD)* was compared to a clinician-led CBT skills group for teens. The results showed that teens using Woebot experienced similar reductions in depressive symptoms, along with high levels of satisfaction and engagement.

Similarly, *Wysa*, an AI-powered app offering CBT-based support, has shown encouraging results in real-world use. A 2022 mixed-methods study involving over 1,200 users found that most developed a strong sense of connection with the app within just a few days. Among users who engaged with Wysa more frequently, many reported meaningful improvements in their mood and symptoms of depression (50). These examples demonstrate how thoughtfully designed AI tools can complement traditional therapy, making mental health support more accessible, especially for youth in rural or underserved communities.

Future developments will likely focus on addressing existing current limitations, such as lack of empathy, risks of misinformation, and safety protocols. Combining AI systems with human oversight and aligning them with clinical standards (17) can improve their safety and effectiveness (17). Emerging techniques in prompt engineering, such as CoT and IPT, are improving the ability of AI tools to guide structured therapeutic conversations, ask meaningful follow-up questions, and respond to user cues. These improvements support emotionally intelligent interactions that enhance rather than replace traditional therapy. Programs aiming to build prompt literacy could include school-based digital health workshops or be embedded within community youth leadership initiatives, giving adolescents and their adult allies tools to guide safe AI use.

## Authors’ perspective

As nurse scientists, we have seen firsthand the profound impact of community-led mental health efforts in rural settings. Nurses are uniquely equipped to lead this work—not only because of their holistic, relationship-based approach to care, but also their ability to operate across clinical, educational, and policy domains. In rural areas, nurse scientists can facilitate community listening sessions, lead digital literacy training for youth and parents, advocate for broadband and infrastructure investment, and co-design culturally relevant interventions. These actions create a bridge between evidence-based care and real-world community needs.

The limitations of traditional models, particularly in mental health, underscore the need for adaptable and digitally enabled solutions. This perspective draws from both empirical literature and direct engagement with youth and healthcare leaders in rural communities. Nurses are uniquely equipped to lead in this space, not only because of their grounding in holistic care but also because of their ability to foster trust across sectors. This manuscript reflects the strong commitment to reimagining youth mental health support systems that are inclusive, sustainable, and empowering.

## Conclusion

To support the responsible and effective use of AI mental health tools for rural adolescents, coordinated, cross-sector collaboration is essential. Policymakers must prioritize rural broadband expansion, develop regulatory frameworks tailored to pediatric AI use, and fund community-based pilot programs with rigorous evaluation. Healthcare systems should focus on integrating AI into existing services, training staff in ethical and effective use, and partnering with schools, faith-based groups, and youth organizations. At the community level, building coalitions, training trusted adults, and advocating for infrastructure support can help create a foundation for success. Researchers have a critical role in evaluating real-world implementation, ensuring equity in study populations, and refining tools for long-term use. While AI holds promise for expanding access and supporting early intervention, its use must be guided by ethical considerations, including data privacy, consent, algorithmic fairness, and digital inclusion. Importantly, AI should enhance—not replace—human connection. For example, school counselors might use AI to support student monitoring while maintaining the therapeutic relationship. By actively involving communities and maintaining a clear focus on equity and trust, we can build mental health systems that are accessible, culturally relevant, and responsive to the unique realities of rural life. This interdisciplinary, community-rooted approach offers a hopeful and actionable path forward.

## Data Availability

The original contributions presented in the study are included in the article/supplementary material, further inquiries can be directed to the corresponding author.
